# Posterior Quadratus Lumborum Block or Thoracolumbar Interfascial Plane Block and Postoperative Analgesia after Spinal Surgery: A Randomized Controlled Trial

**DOI:** 10.3390/jcm12237217

**Published:** 2023-11-21

**Authors:** Şenay Canikli Adıgüzel, Dilan Akyurt, Hatice Bahadır Altun, Gökçe Ültan Özgen, Sevda Akdeniz, Birol Bayraktar, Serkan Tulgar, Yavuz Yiğit

**Affiliations:** 1 Department of Anesthesiology and Reanimation, Faculty of Medicine, Samsun Training and Research Hospital, Samsun University, 55090 Samsun, Turkey; drsenaycanikli@yahoo.com (Ş.C.A.); serkantulgar.md@gmail.com (S.T.); 2 Department of Neurosurgery, Faculty of Medicine, Samsun Training and Research Hospital, Samsun University, 55090 Samsun, Turkey; 3 Department of Emergency Medicine, Hamad Medical Corporation, Doha 3050, Qatar

**Keywords:** lumber disc surgery, thoracolumbar interfascial plane block, posterior quadratus lumborum block, opioid consumption, postoperative pain

## Abstract

Introduction: The management of postoperative pain following lumbar disc herniation (LDH) surgery is crucial for the quality of recovery. The effectiveness of multimodal analgesia plans increases when interfascial plane blocks are included. This study sought to compare the analgesic efficacy of preoperative ultrasound-guided TLIP (thoracolumbar interfascial plane) blocks and posterior QLBs (quadratus lumborum blocks) in patients undergoing LDH surgery. Methods: Patients undergoing elective LDH surgery under general anesthesia were randomized into two groups: thoracolumbar interfascial plane block (Group T) and posterior quadratus lumborum block (Group Q). Block applications were performed 30 min before anesthesia induction. In the postoperative period, analgesia control was provided with a patient-controlled analgesia device. The patients’ 24 h cumulative opioid consumption was examined. Pain scores were evaluated in the 0th, 3rd, 6th, 9th, 12th, and 24th hours. Results: The mean 24 h cumulative morphine consumption for patients was statistically insignificant when Groups T and Q were compared (9.14 ± 7.03 mg vs. 8.66 ± 6.58 mg, *p* = 0.788). Pain scores at rest and during movement as well as morphine consumption were similar between groups in the 0th, 3rd, 6th, 9th, 12th, and 24th hours (*p* > 0.05). Conclusions: The study determined that the utilization of TLIP blocks and posterior QLBs prior to anesthesia induction yielded comparable outcomes in terms of reducing postoperative analgesic consumption and enhancing the efficacy of multimodal analgesia in individuals undergoing single-distance lumbosacral spine surgery under general anesthesia.

## 1. Introduction

Back and leg pain arising from lumbar disc herniations (LDHs) are widespread, and their treatment can range from pharmacotherapy to surgical intervention. Although postoperative pain is less frequent in minimally invasive surgeries when compared to more conventional procedures, it still remains a significant challenge for healthcare providers [1,2]. A majority of patients who undergo spinal surgery report experiencing moderate pain six months after the operation [2].

Following spinal surgery, multimodal analgesia strategies are recommended in order to provide optimal pain management and to promote early functional improvement. These strategies encompass several approaches, including neuraxial blocks, local infiltration analgesia, and fascial plane blocks. Additionally, the utilization of pharmacological medicines such as nonsteroidal agents, opioids, and gabapentinoids is encouraged [3,4]. Interfascial plane blocks, including the thoracolumbar interfascial plane (TLIP) block, erector spinae plane (ESP) block, and quadratus lumborum block (QLB), play a significant role in contemporary medical practice for enhancing post-spinal surgery pain management. These blocks are also employed with the objective of mitigating the adverse effects associated with opioid use, such as nausea, vomiting, allergic reactions, sedation, and respiratory depression [5,6,7,8,9,10].

The ultrasound-guided TLIP block was first described by Hand et al. in 2015 [11], and there are a limited number of studies reporting its effective use for postoperative analgesia in lumbar spine surgery [12]. Blanco et al. [13] introduced the posterior QLB, an interfascial plane block predominantly employed for postoperative analgesia in abdominal surgery [14,15,16], with only a limited number of anecdotal studies documenting its application in lumbar spine surgery.

While the sonographic target is more challenging to identify with the conventional TLIP technique, the anticipated spread of local anesthetic is confined to a narrow area. This circumstance makes the block application more challenging, but it potentially enhances the block’s success. On the other hand, in the posterior QLB technique, the sonographic target is easier to identify, and the area where the local anesthetic will disperse is broader. While this makes the block application easier, it may reduce the sensory coverage of the block. We aimed to compare these two techniques in this study [11,12,14].

The study’s primary objective was to assess and compare the analgesic efficacy of preoperative ultrasound-guided TLIP blocks and posterior QLBs in patients undergoing LDH surgery. We primarily assessed 24 h opioid consumption and secondarily examined postoperative pain scores (NRS) as well as potential opioid-related side effects (e.g., allergies, nausea, vomiting) and complications related to the blocks.

## 2. Materials and Methods

### 2.1. Study Design

This prospective, randomized, and evaluator-blinded study was conducted between August 2022 and August 2023 following ethical board approval (Clinical Research Ethics Committee of the Turkish Ministry of Health, Turkey—Protocol No. 22-AKD-26) and clinicaltrials.gov registration (NCT05421585).

The study comprised individuals aged 18 to 70 who were classified as ASA I-III and were scheduled to undergo single-level lumbar hemilaminectomy/microdiscectomy under general anesthesia. All patients included in the study were operated on with the same surgical technique. The exclusion criteria applied to patients who had a bleeding diathesis, were undergoing anticoagulant therapy, had allergies or hypersensitivity to local anesthetics and opioids, had infections in the block application site, had a history of prior lumbar surgery, had recently used gabapentinoids or corticosteroids within the past three weeks, were unable to cooperate sufficiently to utilize patient-controlled analgesia devices, were suspected to be pregnant, were pregnant or breastfeeding, or had declined to undergo the procedure.

### 2.2. Randomization and Blinding

Upon admission to the room where the preoperative blocks were performed, patients were randomized using the sequentially numbered, opaque, sealed envelope (SNOSE) technique into two groups, each comprising 30 patients (Group Q = posterior QLB group, Group T = classical TLIP block group). The blocks were administered to patients by the same practitioner (ST), and all postoperative assessments were conducted by a blinded anesthetist (DA) to ensure study blindness.

### 2.3. Ultrasound-Guided Block Applications

All block procedures were performed in the block room, 30 min prior to the commencement of surgery. Prior to the block, patients were positioned in the prone position, monitored, and premedicated with 1 milligram (mg) of intravenous (IV) midazolam. Aseptic measures were implemented to maintain sterile conditions, and a lower-frequency ultrasonic transducer (3–5 MHz, Esoate MyLab™30Gold, Genoa, Italy) was appropriately covered with a sterile drape. The study employed an 85 mm block needle (Vygon Echoplex, 85 mm, 21 G, Ecouen, France). The bilateral administration of the blocks involved the use of 20 milliliters (mL) of local anesthetic (%0.25 bupivacaine) for each side.

Posterior QLB application: In contrast to the conventional approach, we deviated from the practice of administering this block in the shamrock or supine position. Instead, we repositioned the transducer from the lumbar midline to a lateral orientation. After achieving visualizations of the spinous processes, erector spinae muscle group, facet joints, transverse processes, and the quadratus muscle in succession, we confirmed the plane by injecting 1–2 mL of physiological saline into the interfascial region located posterior to the quadratus lumborum muscle. Upon the verification of the block’s placement, we proceeded to administer 20 mL of local anesthetic into the interfascial plane. This procedure was then replicated on the contralateral side.

TLIP block application: The ultrasound probe was oriented in a vertical position relative to the L3 vertebra. To aid in positioning, the spinous process and interspinal muscles were identified as reference landmarks. Following this, the longissimus and multifidus muscles were visualized. The block needle was guided in a lateral to medial trajectory using an in-plane approach. To verify the extent of the block, 2 mL of physiological saline was administered in the interfascial space between the longissimus and multifidus muscles. After confirmation, 20 mL of local anesthetic was administered for the execution of the block. The same procedure was replicated on the contralateral side.

### 2.4. Management of General Anesthesia and Perioperative Pain

Patients were transported to the operating room, where they were subjected to monitoring procedures in compliance with ASA standards. Subsequently, the patients were intubated following the administration of 2–2.5 mg/kg^−1^ iv propofol, 1–1.5 microgram/kg^−1^ iv fentanyl, and 0.6 mg/kg^−1^ iv rocuronium. Patients were subsequently positioned in a prone orientation. Anesthesia maintenance was achieved with 0.8–1 MAC (minimal alveolar concentration) sevoflurane in a 50% oxygen–air mixture and 0.1–0.3 mcg/kg/minute (min) iv remifentanil infusion. The lumbar discectomy/laminectomy technique was consistently executed by an identical surgical team, employing a standardized surgical protocol across all patients. In accordance with the routine analgesia protocol, patients were given 1 gram (g) of paracetamol and 20 mg tenoxicam iv approximately 30 min before the end of the surgery as well as 4 mg of iv ondansetron to prevent nausea and vomiting. Patients with adequate spontaneous respiration were extubated and taken to the postoperative recovery unit.

### 2.5. Standard Postoperative Analgesia Protocol and Measurements of Pain

During the postoperative phase, patients received intravenous paracetamol at a dosage of 1 g thrice daily, in addition to intravenous morphine infusion facilitated by patient-controlled analgesia (PCA) devices. The PCA device contained morphine at a concentration of 0.5 mg/mL in normal saline. The device was programmed to administer a bolus dose of 1 mg, with a lockout period of 8 min between doses. Additionally, the device was set with a maximum hourly limit of 6 mg of morphine. The evaluation of postoperative pain was performed using the numeric rating scale (NRS), where a score of 0 represents the absence of pain and a score of 10 signifies the most severe pain imaginable. The NRS was utilized to measure pain levels both at rest and during activity at several time points following the surgical procedure. These time points were the 0th, 3rd, 6th, 9th, 12th, and 24th postoperative hours. The patients were provided with instructions to initiate patient-controlled analgesia (PCA) when their numeric rating scale (NRS) score was ≥4.

### 2.6. Outcome Measurements

The primary outcome of this study was 24 h cumulative opioid requirement as delivered from the PCA device. The secondary outcome was pain score at rest and during movement in the postoperative period. Additionally, we also assessed potential negative outcomes, including allergic responses, nausea, vomiting, and other block-related complications.

### 2.7. Sample Size and Statistical Analysis

Prior to conducting the study, G*Power 3.1.9.7 was utilized to establish the appropriate sample size for equal distribution among the groups. The calculations were based on the following parameters: a significance level (α) of 0.05, a power (1 − β) of 0.80, and an effect size of 0.8. Consequently, it was concluded that a sample size of 26 individuals per group would be necessary. In order to account for potential dropouts, a sample size of 30 patients was selected for each group, resulting in a total of 60 patients. Statistical analyses were conducted using IBM SPSS Statistics 21. The normal distribution of data was assessed by the Kolmogorov–Smirnov and Shapiro–Wilk tests. The independent-samples *t*-test was employed for data that exhibited normal distribution, whereas the Mann–Whitney U-test was favored for data that did not adhere to normal distribution. Chi-square tests were employed to assess the relationships between categorical variables. In addition, mean, standard deviation, median, minimum, maximum; and quartiles (25th and 75th percentiles) were used as distribution measures. A significance level of *p* < 0.05 was deemed to be statistically significant. For NRS scores, the Bonferroni correction was employed in order to account for the multiple comparisons made across the six time periods, and a statistical significance threshold of *p* < 0.0083 was set.

## 3. Results

**Figure 1:**
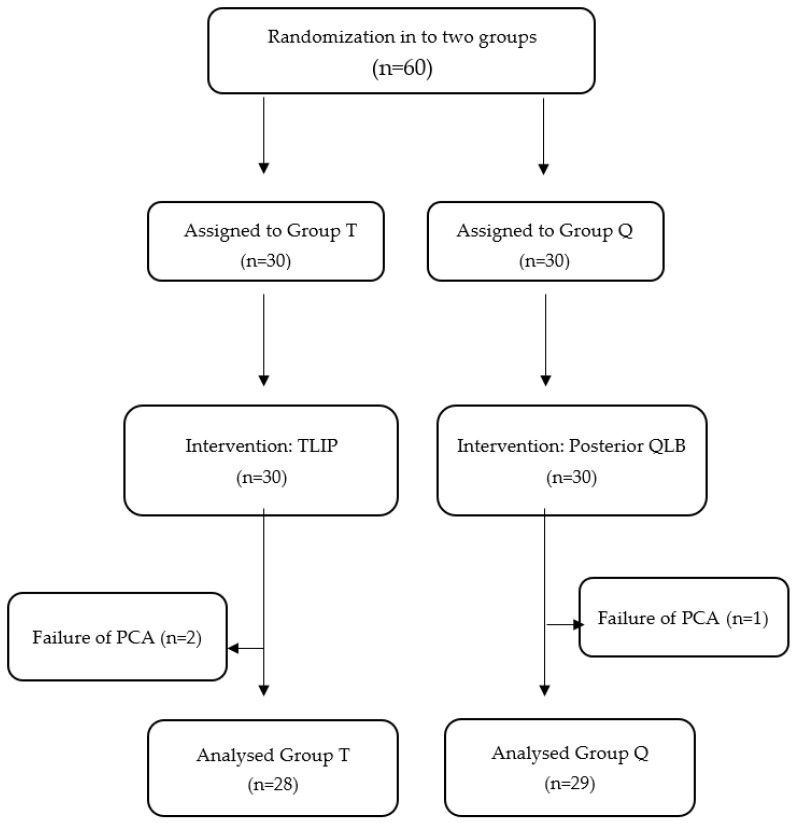
CONSORT flow diagram of study.

The study encompassed a cohort of 60 patients, evenly distributed with 30 patients allocated to each group. However, technical issues related to the PCA device were encountered in two patients in Group T and one patient in Group Q, and consequently the data of these patients were not included in the analysis, as illustrated in the CONSORT diagram of the study (Figure 1). Therefore, the data of 57 patients, with 28 in Group T and 29 in Group Q, were subjected to analysis. The patients’ age, gender, ASA class, BMI values, and operation durations were comparable between the two groups (*p* > 0.05) (Table 1).

**Figure 2:**
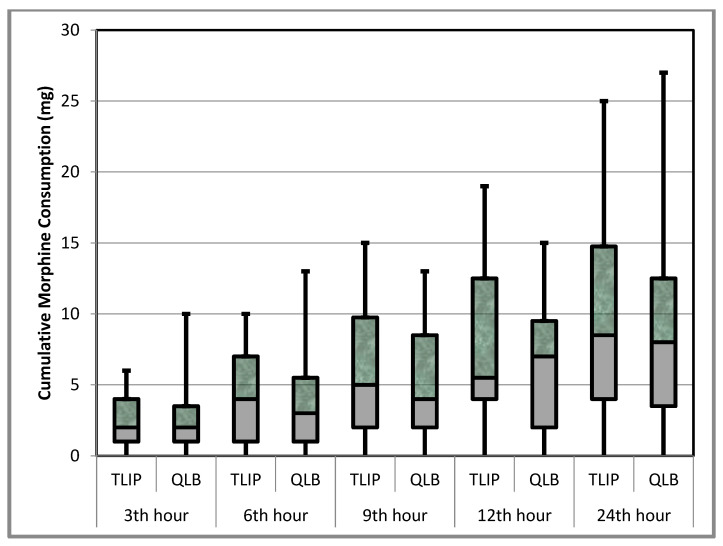
Cumulative morphine consumption (mg) minimum, 1st quarter, median, average, 3rd quarter, and maximum values are shown in the chart.

The mean 24 h cumulative morphine consumption for the patients was similar in both groups (9.14 ± 7.03 mg in Group T vs. 8.66 ± 6.58 mg in Group Q, *p* = 0.788). The morphine consumption at the 0th, 3rd, 6th, 9th, 12th, and 24th hours was also similar between the groups (*p* > 0.05) (Table 2 and Figure 2). The pain scores of the patients during movement and at rest were similar for both groups in the 0th, 3rd, 6th, 9th, 12th, and 24th hours (*p* > 0.05) (Table 3). No block-related complications were observed in any patients. Nausea was reported in three patients in Group T and one patient in Group Q. Quadriceps weakness was not detected in any patients.

## 4. Discussion

Our study demonstrated that the use of US-guided TLIP and posterior QLB techniques as part of multimodal analgesia in patients undergoing single-level lumbar disc surgery resulted in comparable postoperative opioid requirements across all time frames. Furthermore, the NRS scores at rest and during movement were similar at all measured time points. Both groups’ postoperative pain levels remained within acceptable limits.

Lumbar disc herniation surgeries are procedures notably associated with postoperative pain [1,3]. The implementation of efficient strategies for managing postoperative pain has been shown to result in faster mobilization, increased patient satisfaction, and a decrease in complications [17,18,19]. In addition to systemic opioids, central neuraxial blocks and paravertebral blocks have been employed for postoperative pain relief. Unfortunately, it has been reported that around 9% of patients continue using opioids a year after undergoing spinal surgery [12]. Interfascial plane blocks, a component of multimodal analgesic regimens, are suitable for optimizing post-spinal surgery pain relief and minimizing opioid-related adverse effects.

In our study, we augmented our multimodal analgesia strategy by incorporating fascial plane blocks to establish an effective analgesic protocol for our patients. To achieve this goal, we employed the recently introduced TLIP block and QLB, assessing the efficacy of postoperative pain relief.

The erector spinae muscle group comprises three muscles located in the lumbar region: the multifidus, longissimus, and iliocostalis, collectively known as the paraspinal muscles. The primary fascia in the lumbar region, known as the thoracolumbar fascia, envelops the erector spinae, quadratus lumborum, psoas major, and latissimus dorsi muscles. The thoracolumbar fascia is composed of three layers: anterior, posterior, and middle (alper).

In the initial classical form of the TLIP block, a local anesthetic is administered into the plane situated between the multifidus and longissimus muscles, selectively targeting the dorsal ramus and medial branch [9,11]. Subsequently, this technique underwent modification by Ahıskalıoglu et al. [20,21]. In the modified TLIP block, the local anesthetic is administered into the anatomical plane situated between the longissimus and iliocostalis muscles, with the intention of selectively affecting the corresponding nerves. The authors claim that the current approach simplifies the process of sono-anatomical identification [19,20]. In our study, we employed the classical TLIP technique and encountered greater difficulty in sono-anatomical identification.

The existing body of literature on the application of TLIP blocks using the classical method in patients undergoing lumbar disc surgery remains limited [8,22]. Nonetheless, there exists moderate-quality evidence indicating that TLIP blocks are efficacious in the management of postoperative pain subsequent to lumbar spine surgery. This intervention leads to diminished pain scores during periods of both rest and movement for up to 24 h. Additionally, TLIP blocks contribute to a reduction in overall analgesic usage and the risk of postoperative nausea and vomiting (PONV) [22]. In a meta-analysis [23] assessing TLIP blocks (classical and modified) in patients undergoing spinal surgery, the analgesic efficacy of TLIP blocks was reported to surpass that of wound infiltration. Additionally, Chen et al. [24] demonstrated a reduced postoperative use of sufentanil in patients who received a TLIP block during spine surgery.

In the posterior quadratus lumborum block (QLB), local anesthetic is administered in a plane immediately lateral to that utilized in the erector spinae plane block (ESPB) [5,13,25]. This technique effectively blocks the lateral and anterior cutaneous branches of the ilioinguinal, iliohypogastric, and subcostal nerves, which innervate the lower abdominal region as well as the paraspinal region by extending posteriorly and blocking branches of the dorsal ramus [9,26,27,28]. The QLB can be performed through three distinct approaches: anterior, posterior, and lateral. In our study, we opted for the posterior approach. In a study conducted by Sertcakalılar et al. [28], the posterior QLB approach was employed in patients undergoing lumbar disc surgery, resulting in lower postoperative pain scores at all time intervals and a significant reduction in morphine consumption at both 2 and 6 h postoperatively [28]. Additionally, Wilton et al. [25] reported a significantly decreased opioid consumption in patients who underwent lumbar spinal surgery and received continuous quadratus lumborum block catheterization for postoperative analgesia.

To the best of our knowledge, this study is the first to compare the efficacy of the TLIP and posterior QLB techniques in the context of single-level lumbar spinal surgeries. While there are a limited number of similar studies utilizing these blocks in the existing literature, our investigation yielded noteworthy findings. Specifically, we observed a statistically significant reduction in 24 h opioid consumption within both the TLIP and QLB groups, and the levels of opioid utilization were found to be comparable between these groups. It is important to note that although we did not incorporate a control or sham group into our study design, the relatively low 24 h opioid consumption observed in our patient cohort, particularly in light of the absence of any scheduled analgesic beyond paracetamol in our postoperative analgesia regimen, is a notable outcome when juxtaposed with the existing literature [8,22].

Our study was subject to several limitations. Firstly, we did not conduct a dermatome analysis or measure the sensory block area, which could be perceived as a constraint. However, it is worth noting that these assessments were not included in similar studies, primarily due to the nature of the surgical procedures involved, which are not conducive to such analyses. Additionally, we did not record the block performance times, although our clinical observations suggested that the identification process was quicker with the quadratus lumborum block (QLB).

Another limitation was that, contrary to the practice of some anesthetists, we implemented all blocks in the preoperative block performance room, even though they could have been carried out in the prone position after induction. Our aim with this approach was to shorten the operating room usage time and identify potential early complications, and we hypothesized a certain time requirement for the local anesthetic to reach the target tissue. In addition to studies investigating the effects of different blocks, research exploring the pros and cons of the two time options could also be beneficial.

Furthermore, we did not assess postoperative patient satisfaction scores or quality of recovery scores, both of which should unquestionably be included in future investigations. Additionally, the somatosensory evoked potential (SSEP) and motor evoked potential (MEP) techniques, recently employed to mitigate nerve damage in lumbar surgeries, could potentially be utilized, although it is important to note that they are not standard practice for single-level disc surgeries. Nevertheless, it is advisable to consider future research endeavors aimed at determining the potential impact of the TLIP and QLB techniques on the necessity of monitoring techniques in thoracolumbar spinal surgeries.

## 5. Conclusions

We determined that the TLIP and posterior QLB techniques applied before anesthesia induction similarly reduced the postoperative analgesic requirement and improved the quality of multimodal analgesia in patients undergoing single-distance lumbosacral spine surgery under general anesthesia.

## Figures and Tables

**Table 1:** Demographical and clinical data for both groups.

	Group T (n = 28)	Group Q (n = 29)	*p*
**Age (years)**	43.07 ± 14.04	42.79 ± 11.70	0.935
**Gender (male/female)**	17/11	16/13	0.877
**ASA I/II/III (n)**	6/21/1	11/16/2	0.292
**Operation duration (minutes)**	87.43 ± 28.38	86.72 ± 20.15	0.968
**Level of surgery (L2–L3/L3–L4/L4–L5/L5–S1) (n)**	2/14/11/1	1/15/13/0	
**BMI (kg/m^2^)**	26.22 ± 3.05	26.64 ± 2.87	0.594

n = number, ASA = American Society of Anesthesiologists, BMI = body mass index.

**Table 2:** Opioid consumption between Groups T and Q at different time periods.

Morphine Consumption (mg)	Group T (n = 28)	Group Q (n = 29)	*p*
**0th hour**	0 (0–0)	0 (0–0)	0.384
**3rd hour**	2 (1–4)	2 (1–3.5)	0.948
**6th hour**	4 (1–7)	3 (1–5.5)	0.403
**9th hour**	5 (2–9.75)	4 (2–8.5)	0.516
**12th hour**	5.5 (4–12.5)	7 (2–9.5)	0.575
**24th hour**	8.5 (4–14.75)	8 (3.5–12.5)	0.780

Since data were not normally distributed, they are expressed as the median (percentile 25–75).

**Table 3:** Comparison of NRS scores between groups T and Q.

	Group T (n = 28)	Group Q (n = 29)	*p*
**NRS at rest**			
**0th hour**	2.5 (0–3)	2 (0–2.5)	0.283
**3rd hour**	2 (1.25–3)	2 (1.5–2.5)	0.496
**6th hour**	2 (1–2)	2 (1–2)	0.635
**9th hour**	1 (1–2)	2 (1–2)	0.556
**12th hour**	1 (1–2)	1 (1–2)	0.873
**24th hour**	1 (1–2)	18 (1–2)	0.937
**NRS in motion**			
**3rd hour**	3 (2–4)	3 (2–3)	0.724
**6th hour**	2 (2–3)	3 (2–3)	0.342
**9th hour**	2 (2–3)	2 (2–3)	0.110
**12th hour**	2 (2–3)	2 (2–3)	0.432
**24th hour**	2 (1.25–2)	2 (1.5–2)	0.390

NRS = numeric rating scale, n = number.

## Data Availability

The data presented in this study are available on request from the corresponding author. The data are not publicly available due to patient privacy protection.
